# Postoperative Pain Management in Total Knee Arthroplasty

**DOI:** 10.1111/os.12535

**Published:** 2019-10-29

**Authors:** Jing‐wen Li, Ye‐shuo Ma, Liang‐kun Xiao

**Affiliations:** ^1^ Department of Orthopaedic Surgery Yueyang Second People's Hospital Yueyang China; ^2^ Department of Orthopaedic Surgery Yueyang Hospital Affiliated to Hunan Normal University Yueyang China; ^3^ Xiangya School of Pharmaceutical Sciences Central South University Changsha China

**Keywords:** Knee joint pain, Multimodal analgesia, Postoperative pain, Total knee arthroplasty

## Abstract

Total knee arthroplasty (TKA) is one of the most common surgeries performed to relieve joint pain in patients with end‐stage osteoarthritis or rheumatic arthritis of the knee. However, TKA is followed by moderate to severe postoperative pain that affects postoperative rehabilitation, patient satisfaction, and overall outcomes. Historically, opioids have been widely used for perioperative pain management of TKA. However, opioids are associated with undesirable adverse effects, such as nausea, respiratory depression, and retention of urine, which limit their application in daily clinical practice. The aim of this review was to discuss the current postoperative pain management regimens for TKA. Our review of the literature demonstrated that multimodal analgesia is considered the optimal regimen for perioperative pain management of TKA and improves clinical outcomes and patient satisfaction, through a combination of several types of medications and delivery routes, including preemptive analgesia, neuraxial anesthesia, peripheral nerve blockade, patient‐controlled analgesia and local infiltration analgesia, and oral opioid/nonopioid medications. Multimodal analgesia provides superior pain relief, promotes recovery of the knee, and reduces opioid consumption and related adverse effects in patients undergoing TKA.

## Introduction

Total knee arthroplasty (TKA) is commonly performed in patients with end‐stage osteoarthritis or rheumatic arthritis of the knee to relieve joint pain, increase mobility, and improve quality of life. However, TKA is followed by moderate to severe postoperative pain^1^. In patients received TKA, 60% experience severe postoperative knee pain and 30% experience moderate pain^2^. Some patients even put off this operation because of the fear of this acute postoperative pain^3^. Furthermore, postoperative pain in TKA inhibits early ambulation and range of motion, risking thromboembolism, and affects rehabilitation, patient satisfaction, and overall outcomes.

**Figure 1:**
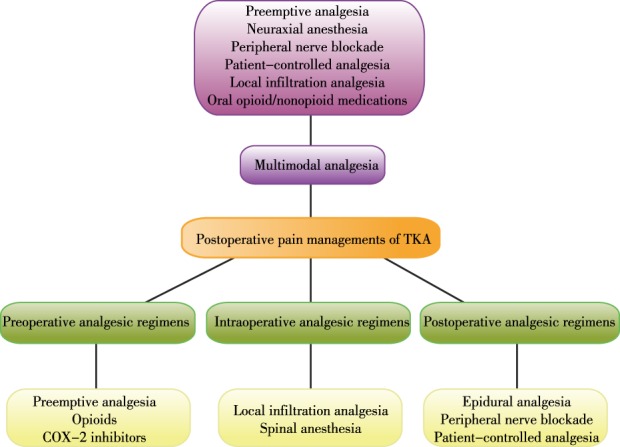
Commonly used postoperative pain management regimes for total knee arthroplasty (TKA). COX‐2, cyclooxygenase‐2.

In 1996, the American Pain Society declared that pain was “the fifth vital sign”^3^. In an attempt to relieve severe postoperative pain, several routine approaches have been proposed, such as use of preemptive analgesia, opioids, cyclooxygenase‐2 inhibitors, epidural anesthesia, peripheral nerve blockade, local infiltration analgesia, patient‐controlled analgesia, and multimodal analgesia. Adequate postoperative analgesia could not only reduce pain, opioid consumption, and, consequently, opioid‐related adverse events, but also reduce length of hospital stay and costs, and improve rehabilitation and patient satisfaction^4^. Therefore, it is necessary for surgeons to fully understand current anesthetic and analgesic regimens for TKA to improve patient outcomes (Fig. 1).

## Materials and Methods

**Figure 2:**
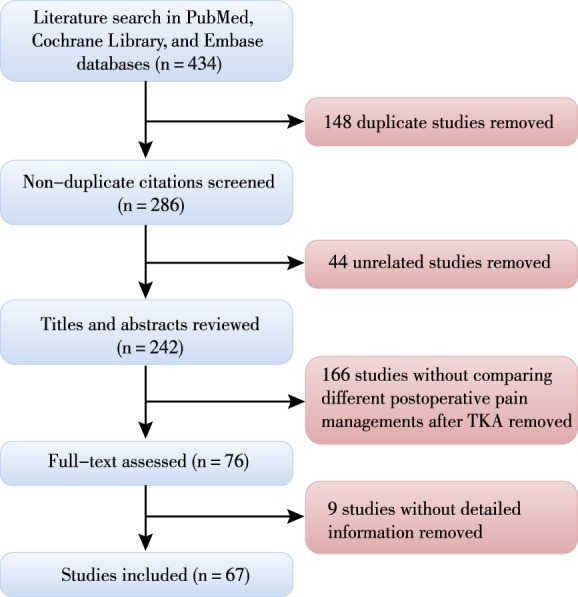
Flow chart of the search for published reports showing the process of inclusion and exclusion. TKA, total knee arthroplasty.

A review of the available literature was performed on 8 January 2019 through searching PubMed, Cochrane Library and EMBASE databases. The keyword terms “pain management,” “pain control,” and “total knee arthroplasty” were used for searching the literature. The titles, abstracts, and full texts of published studies were screened. As a result, 67 studies were included in this review. This entire process is depicted in Fig. 2.

## Multimodal Analgesia

With exploration of the mechanisms underlying postoperative pain in TKA, it has been verified that both peripheral and central mechanisms are involved. Therefore, monotherapy alone is not enough to provide satisfactory postoperative pain relief after TKA. At present, multimodal analgesia is considered as the optimal method for perioperative pain management of TKA through targeting numerous pain pathways. Multimodal analgesia was first introduced by Wall in 1988, referring to a combination of several types of medications and delivery routes, including preemptive analgesia, neuraxial anesthesia, peripheral nerve blockade (PNB), patient‐controlled analgesia (PCA), local infiltration analgesia (LIA), and oral opioid and nonopioid medication^5^.

Multimodal analgesia includes preoperative, intraoperative, and postoperative analgesic regimens, aiming to maximize the analgesic efficacy through the combination of several analgesic regimens, while minimizing undesired adverse effects. Adequate preemptive analgesia could prevent pain nociceptors from entering a state of hyperalgesia, and make acute postoperative pain easier to control, ultimately reducing opioid consumption. Intraoperatively, LIA is performed by a surgeon near the conclusion of a procedure that directly prevents the generation and conduction of pain signals from incision. Several medicines are available for LIA during the surgery, which will be discussed in detail later. Postoperatively, multimodal analgesia includes pharmacologic agents, neuraxial anesthesia, PNB, and PCA, each of which will be described below. Compared with a monotherapy, multimodal analgesia provides superior postoperative pain relief to promote recovery of the knee, and reduce opioid consumption and related side effects^1^, ^4^. Consequently, multimodal analgesia is used widely for perioperative pain control in patients undergoing TKA.

## Preoperative Analgesic Regimens

### *Preemptive Analgesia*

Preemptive analgesia is defined as an antinociceptive intervention that starts before a surgical procedure. It is more effective than the same intervention when started after surgery^6^. Preemptive analgesia is intended to prevent peripheral and central hypersensitivity, decrease the incidence of hyperalgesia, and reduce the intensity of postoperative pain^6^, ^7^. Preemptive analgesia also increases the pain threshold, contributing to lower postoperative application of analgesic medication.

Cyclooxygenase‐2 (COX‐2) inhibitors, such as parecoxib sodium and celecoxib, administered 30–60 min before surgery significantly decrease postoperative pain and morphine consumption without increasing the incidence of other postoperative complications^8^, ^9^. Parecoxib sodium 40 mg administered 30 min before surgery significantly decreased postoperative pain in a post‐anesthesia care unit without increasing the incidence of other postoperative complications^8^. Patients administered with celecoxib 400 mg within 1 h before surgery had less pain at rest and walking during the first week postoperatively, and received less morphine at 48 h after the procedure^9^. Lubis *et al*. (2018)^10^ demonstrated that a combination of pregabalin (an antiallodynic and antihyperalgesic) and celecoxib could be used as preemptive analgesia in TKA through their synergic effects. Enrolled patients were given celecoxib 400 mg and pregabalin 150 mg 1 h before the operation, or celecoxib 200 mg and pregabalin 75 mg twice daily starting from 3 days before the operation, or a placebo. The results showed that preemptive analgesia with celecoxib and pregabalin was effective in reducing postoperative usage of morphine independent of the dosage. Another investigation by Xu *et al*.^11^ evaluated the efficacy of combination of celecoxib (200 mg, BID) and tramadol/acetaminophen (37.5 mg/325 mg, TID) 3 days preoperatively in treating postoperative pain of patients undergoing unilateral TKA. It verified that this regimen of preemptive analgesia significantly decreased resting pain at 3 weeks and 6 weeks, and movement pain at 1/3/6 weeks and 3 months postoperatively^11^. Therefore, preemptive analgesia is an important component in multimodal analgesia to reduce postoperative analgesia usage.

### *Opioids*

Opioids have long been used for the treatment of knee arthritis and perioperative pain management of patients undergoing primary TKA and revision knee arthroplasty. As analgesics, opioids inhibit the conduction of pain signals through activation of opioid receptors through several delivery methods, such as oral, intravenous, intramuscular, subcutaneous, and transdermal. Although opioids are effective in pain management after TKA, there are some adverse effects associated with them, such as itchiness, nausea, somnolence, respiratory depression, retention of urine, and constipation. In addition, long‐term use of opioid medications may lead to tolerance and dependence that require increasing doses to achieve the same effects. Therefore, it is practical to apply other analgesic regimens to reduce the amount of opioid consumption.

Bedard *et al*. (2017) suggested that approximately one‐third of TKA patients used opioids within 3 months prior to surgery^12^. Compared with total hip arthroplasty patients, TKA patients were twice as likely to require refill opioid prescriptions and were prescribed a greater total morphine equivalent dose for a longer period of time postoperatively^13^. Increasing evidence reveals that preoperative chronic use of opioids reduces the effect of pain relief postoperation, and increases postoperative opioid consumption in TKA patients^12^, ^14^, ^15^, ^16^. In addition, preoperative opioid use is associated with early revision, postoperative complications, worse clinical outcomes due to developed tolerance, and hyperalgesia, which can complicate recovery and rehabilitation^17^, ^18^, ^19^. These results suggest that limiting preoperative opioid use can optimize the benefits of TKA. Furthermore, Nguyen *et al*.^20^ evaluated whether weaning of opioid use (50% reduction in morphine‐equivalent dose) in the preoperative period improved total joint arthroplasty outcomes. Their results showed that the patients successfully weaned before surgery had substantially improved clinical outcomes, including for The Western Ontario and McMaster Universities Arthritis Index, SF12v2, and the University of California at Los Angeles activity score, which were comparable to patients who did not use opioids at all. This study highlights the importance of reduction of preoperative opioid use to improve postoperative recovery in TKA patients.

### *Cyclooxygenase‐2 Inhibitors*

Traditional nonsteroidal anti‐inflammatory drugs (NSAID) and COX‐2 inhibitors provide perioperative analgesia through inhibition of COX‐2 and prostaglandins. NSAID also inhibit the activity of COX‐1 that is associated with gastrointestinal effects. In addition, NSAID increase the risk of perioperative blood loss. Fortunately, COX‐2 inhibitors have a favorable adverse effect profile with reduced gastrointestinal effects and risk of blood loss^21^. COX‐2 inhibitors exert the effect of analgesia through reducing the synthesis of peripheral prostaglandin to relieve inflammation and inhibit peripheral and central COX‐2 expression, ultimately preventing the sensitization of central nerve system.

A meta‐analysis conducted by Lin *et al*.^22^ with 571 patients showed that perioperative administration of selective COX‐2 inhibitors could reduce pain and opioid consumption in patients undergoing TKA, without increasing the risk of blood loss. Furthermore, a randomized controlled trial (RCT) by Munteanu *et al*.^23^ of 165 patients after TKA suggested that preoperative administration of etoricoxib 120  mg orally was superior to postoperative administration of the same dose in terms of its morphine‐sparing effect during the first postoperative 48  h, without difference in the incidence of side effects. Apart from analgesia, celecoxib and parecoxib could also decrease early postoperative cognitive dysfunction incidence after TKA in elderly patients^24^, ^25^. At present, COX‐2 inhibitors are usually added to multimodal analgesia to reduce the consumption of opioids, without serious complications^8^.

## Intraoperative Analgesic Regimens

### *Local Infiltration Analgesia*

Local infiltration analgesia (LIA) has received increasing interest in recent years because of the associated low risk, simple performance, low complication rates, and reduced local anesthetic systemic toxicity. LIA is performed by a surgeon intraoperatively without specialist equipment, commonly near the conclusion of a procedure. Local anesthetic combined with opioids, antibiotics, NSAID or epinephrine (cocktail) are injected into periarticular regions, including the posterior capsule, collateral ligaments, capsular incision, the quadriceps tendon, and subcutaneous tissues, which directly prevents the generation and conduction of pain signals from incision. Recently, LIA has become an alternative analgesic regimen to femoral nerve block (FNB) without resulting in impairment of quadriceps muscle strength. Nevertheless, there is still no consensus on the optimal composition and infiltration technique of LIA. Common LIA cocktails are listed in Table 1.

**Table 1:** Common local infiltration analgesia cocktails

Year	Authors	Components
2017	Wall *et al*.^29^	150 mg 0.25% levobupivacaine hydrochloride, 10 mg morphine sulphate, 30 mg ketorolac trometamol, and 0.25 mg adrenaline diluted with saline to a total volume of 150 mL
2018	Tong QJ *et al*.^65^	150 mg ropivacaine, 30 mg ketorolac, 10 mg morphine, and 200 mcg adrenaline in a total volume of 75 mL
2018	Mont MA *et al*.^35^	266 mg/20 mL liposomal bupivacaine admixed with bupivacaine HCl 0.5%, 20 mL diluted with saline to a total volume of 120 mL
2019	McCarthy D *et al*.^66^	2 mg/ kg levobupivacaine and 0.5 mg adrenaline diluted with saline to a total volume of 100 mL
2019	Koniuch KL *et al*.^67^	30 mg ketorolac, 80 μg clonidine, 0.5 mg epinephrine and weight‐based dosing of ropivacaine (270 mg for patients weighing 80 kg or more)

Among the patients undergoing TKA, those patients who received intraoperative LIA achieved improvement in pain scores and comparable satisfaction levels, with reduced total narcotic consumption in the early postoperative period compared to those with placebo infiltration^26^, ^27^. FNB is widely considered as an effective postoperative pain management protocol. A meta‐analysis conducted by Zhang LK *et al*.^28^, including 10 studies with 950 patients, showed that LIA was as effective as FNB in terms of visual analog scale score for pain control at 24/48/72 h, total morphine consumption, range of motion, knee society score, complications, and length of hospital stay. Wall *et al*.^29^ performed an RCT and demonstrated that the patients with LIA used less morphine in the first postoperative day after TKA compared with the patients with FNB. Another meta‐analysis conducted by Hu *et al*.^30^ with 1206 patients showed that LIA provided superior postoperative pain relief at rest and preserved quadriceps function in the early postoperative period compared with epidural analgesia/peripheral nerve block. These results suggest that LIA is a viable and safe alternative to FNB for postoperative pain management after TKA without impairment of quadriceps muscle function. Furthermore, a meta‐analysis conducted by Sardana V *et al*.^31^ showed that LIA could significantly improve postoperative pain and opioid consumption when compared with adductor canal block (ACB).

Liposomal bupivacaine (LB) is used as a component of the LIA cocktail to prolong the effect of LIA, for LB can last up to 72 hours. Several studies have assessed the efficacy of LIA with LB in patients undergoing TKA, but the results are controversial^32^, ^33^, ^34^. Mont *et al*. (2018)^35^ performed an RCT to compare the effects of LIA, with or without LB, on postsurgical pain control by minimizing the limitations that may have affected previous study results. Their results showed that LIA with LB improved postsurgical pain, opioid consumption (18.7 mg *vs* 84.9 mg, *P* = 0.0048), and time to first opioid rescue without side effects^35^. Although another study showed that patients in an FNB group had significantly lower pain scores (mean 3.24 *vs* 3.87, *P* = 0.02) and higher range of motion (84.54° *vs* 78°, *P* < 0.001) in the first 24 h, the patients receiving LIA with LB were significantly more likely to perform a straight leg raise 12 h postoperatively (73% *vs* 50%; *P* = 0.0003) and scored better in the physical function component of the Short Form‐12 (−23 *vs* −27, *P* = 0.01) 3 months postoperatively^36^. This suggested that LIA with LB provided excellent pain relief that was not inferior to FNB in patients undergoing TKA.

Different results of efficacy of LIA compared with other analgesia protocols are obtained from different studies, which reach no consensus in optimal composition and infiltration techniques of LIA. A previous study suggested that it was intraoperative periarticular instead of intraarticular infiltration that was effective in pain control in the early postoperative period after TKA^37^. Therefore, future research should focus on the optimal composition and infiltration technique of LIA to further verify the efficacy.

### *Spinal Anesthesia*

Anesthesia choice is an issue for better perioperative outcomes after TKA. In addition, pain management after TKA is affected by the type of anesthesia used for the surgery. Both general and spinal anesthesia are appropriate for TKA. Because general anesthesia is associated with reduced perioperative tissue oxygen tension as well as postoperative nausea, vomiting, and delirium, it is initially considered the gold standard for hip and knee arthroplasty^38^, ^39^. However, spinal anesthesia is becoming popular in TKA. Compared with general anesthesia, spinal anesthesia is reported to be associated with a lower rate of superficial wound infections (0.68% *vs* 0.92%, *P* = 0.0003), blood transfusions (5.02% *vs* 6.07%, *P* = 0.0086), length of surgery (96 *vs* 100 min, *P* < 0.0001), and length of hospital stay (3.45 *vs* 3.77 days, *P* < 0.0001)^40^. Furthermore, patients administered general anesthesia have a small but significant increase in the risk of complications^40^. Another investigation conducted by Park *et al*.^41^ showed similar results. They found that patients that received TKA under general anesthesia had longer preoperative room time (+9.4 minutes, *P* < 0.001), postoperative room time (+12.7 min, *P* < 0.001), and postoperative hospital stay (+2.5 days, *P* = 0.001), and had more surgical site infections (5 [1%] *vs* 0 [0%], *P* = 0.005) and blood transfusions (205 [41.8%] *vs* 262 [35.1%], *P* = 0.01) compared to those with spinal anesthesia. However, there were no differences in operative duration and other adverse events. In addition, Mahan *et al*.^42^ (2018) reported that patients who underwent TKA with mepivacaine for spinal anesthesia had a shorter length of stay (28.1 ± 11.2 *vs* 33.6 ± 14.4 h, *P* = 0.002), fewer episodes of straight catheterization (3.8% *vs* 16.5%, *P* = 0.021) compared to bupivacaine, and exhibited no differences in postoperative pain or morphine consumption.

## Postoperative Analgesic Regimens

### *Epidural Analgesia*

Formerly, epidural analgesia was used as a regular postoperative analgesic regimen for patients after TKA, consisting of a local anesthetic agent and an opioid. Compared with parenteral opioids, epidural analgesia provides better postoperative analgesia with less nausea, vomiting, and pruritus^43^. However, some studies show that epidural anesthesia is associated with many adverse effects, such as urinary retention, hypotension, pruritus, and motor block^44^, ^45^, ^46^. Among these, the main drawback of epidural anesthesia is inadvertent motor nerve block, which delays physical therapy and rehabilitation^44^. In addition, epidural anesthesia limits the options for postoperative prophylaxis against deep venous thrombosis. A meta‐analysis by Li *et al*. (2018) of seven RCT, with 251 patients undergoing TKA, concluded that LIA was as effective as epidural anesthesia for pain control^47^. LIA showed an increase in the range of motion, and a reduction of the occurrence of nausea and length of hospital stay^47^. Another meta‐analysis compared analgesic efficacy and side effect profile of FNB and epidural anesthesia through analyzing eight RCT with 510 patients^48^. The results suggested that FNB provided a comparable postoperative analgesia but had a favorable adverse effect profile with less neuraxial complications. Hypotension and urinary retention occurred more frequently among patients receiving epidural anesthesia, with worse patient satisfaction^48^. These studies suggest that FNB and LIA may be preferable to epidural analgesia for postoperative pain relief after TKA.

### *Peripheral Nerve Blockade*

Peripheral nerve blockade is usually used to relieve postoperative pain of TKA. PNB could significantly reduce consumption of opioid and opioid‐related adverse effects. It also promotes early mobilization and reduces the length of hospital stay. The knee joint is innervated by several nerves, including the femoral nerve, the sciatic nerve, the obturator nerve, the saphenous nerve, and the lateral femoral cutaneous nerve. Among these, the femoral nerve is an important one with respect to analgesic effects after TKA. Consequently, FNB is one of the most commonly used PNB, and has been widely accepted as the gold standard for pain relief after TKA. FNB not only provides excellent pain management after TKA but also reduces opioid consumption, hospital stay, and incidence of nausea and vomiting^49^, ^50^. Besides, FNB contributes to long‐term functional recovery in patients undergoing TKA^49^. Although FNB could provide effective postoperative analgesia, it is also associated with some serious complications. It may damage adjacent major blood vessels and nerves itself^50^, and reduces quadriceps muscle strength, which limits extension of the knee and increases risk of falls postoperatively^51^, ^52^. Consequently, ACB is an alternative analgesic regimen to FNB^52^.

The adductor canal is located in the middle one‐third of the thigh and runs from the apex of the femoral triangle proximally to the adductor hiatus distally^2^. ACB could block the saphenous nerve, which is the largest sensory branch of the femoral nerve to the knee, while spare the major motor branches of the femoral nerve. Therefore, ACB could provide postoperative pain relief as effectively as FNB without impairment of quadriceps muscle strength, and it is becoming increasingly popular^2^, ^52^. Except for better quadriceps muscle power, patients with ACB have better early rehabilitation, longer ambulation distance, and shorter length of hospital stay compared with FNB^53^, ^54^. Previous study also suggested that continuous ACB was superior to a single shot block in terms of pain control but was similar for early functional recovery^55^. Nevertheless, ACB is still a newly developed method of regional anesthesia after TKA, and large RCT are needed to further evaluate its application in surgery of the knee.

### *Patient‐controlled Analgesia*

Patient‐controlled analgesia is widely used for pain management in patients after TKA to provide simple, fast, and adequate pain relief without a specialized anesthesiologist postoperatively. The device is programmed according to the analgesic used, the physical characteristics, and the baseline pain of the patients. A small amount of analgesic could be delivered by the patients pressing the button when they most need it. Usually, opioids are used in PCA, such as oxycodone, morphine, fentanyl, and hydromorphone^56^, ^57^, ^58^, ^59^. Therefore, PCA is associated with some adverse effects caused by opioids, including nausea, vomiting, respiratory depression, and urinary retention^60^. However, these adverse effects are less severe than those caused by conventional opioid treatment.^61^ PCA is safe and effective for treating moderate to severe pain and has become increasingly popular for use in patients undergoing TKA^61^, ^62^. Currently, opioid drugs are commonly administered by PCA, adding to multimodal analgesia^1^, ^63^, ^64^.

## Summary

With the aging of the population, there will be an increasing number of elderly patients receiving TKA. Perioperative pain management in patients undergoing TKA is very important to improve rehabilitation, patient satisfaction, and overall outcomes. Furthermore, when surgeons make a plan for perioperative pain management after TKA, they must consider each patient individually. At present, multimodal analgesia is the optimal analgesic regimen for TKA. Multimodal analgesia could improve perioperative pain control and patient satisfaction through the combination of several analgesic regimens, while reducing opioid consumption and opioid‐related adverse effects. However, the optimal protocol of multimodal analgesia needs to be further investigated in the future.

## References

[1] Aso K , Izumi M , Sugimura N , *et al* Additional benefit of local infiltration of analgesia to femoral nerve block in total knee arthroplasty: double‐blind randomized control study. Knee Surg Sports Traumatol Arthrosc, 2019, 27: 2368–2374. 30536047 10.1007/s00167-018-5322-7

[2] Seo SS , Kim OG , Seo JH , Kim DH , Kim YG , Park BY . Comparison of the effect of continuous femoral nerve block and adductor canal block after primary total knee arthroplasty. Clin Orthop Surg, 2017, 9: 303–309. 28861197 10.4055/cios.2017.9.3.303 PMC5567025

[3] Gaffney CJ , Pelt CE , Gililland JM , Peters CL . Perioperative pain management in hip and knee arthroplasty. Orthop Clin North Am, 2017, 48: 407–419. 28870302 10.1016/j.ocl.2017.05.001

[4] Dimaculangan D , Chen JF , Borzio RB , Jauregui JJ , Rasquinha VJ , Maheshwari AV . Periarticular injection and continuous femoral nerve block versus continuous femoral nerve block alone on postoperative opioid consumption and pain control following total knee arthroplasty: randomized controlled trial. J Clin Orthop Trauma, 2019, 10: 81–86. 30705537 10.1016/j.jcot.2017.09.012 PMC6349676

[5] Moucha CS , Weiser MC , Levin EJ . Current strategies in anesthesia and analgesia for total knee arthroplasty. J Am Acad Orthop Surg, 2016, 24: 60–73. 26803543 10.5435/JAAOS-D-14-00259

[6] Pogatzki‐Zahn EM , Zahn PK . From preemptive to preventive analgesia. Curr Opin Anaesthesiol, 2006, 19: 551–555. 16960490 10.1097/01.aco.0000245283.45529.f9

[7] Grape S , Tramèr MR . Do we need preemptive analgesia for the treatment of postoperative pain? Best Pract Res Clin Anaesthesiol, 2007, 21: 51–63. 17489219 10.1016/j.bpa.2006.11.004

[8] Bian YY , Wang LC , Qian WW , *et al* Role of parecoxib sodium in the multimodal analgesia after total knee arthroplasty: a randomized double‐blinded controlled trial. Orthop Surg, 2018, 10: 321–327. 30485685 10.1111/os.12410 PMC6594467

[9] Jianda X , Yuxing Q , Yi G , Hong Z , Libo P , Jianning Z . Impact of preemptive analgesia on inflammatory responses and rehabilitation after primary total knee arthroplasty: a controlled clinical study. Sci Rep, 2016, 6: 30354. 27578313 10.1038/srep30354 PMC5005994

[10] Lubis AMT , Rawung RBV , Tantri AR . Preemptive analgesia in total knee arthroplasty: comparing the effects of single dose combining celecoxib with pregabalin and repetition dose combining celecoxib with Pregabalin: double‐blind controlled clinical trial. Pain Res Treat, 2018, 2018: 3807217. 30174951 10.1155/2018/3807217 PMC6106806

[11] Xu Z , Zhang H , Luo J , Zhou A , Zhang J . Preemptive analgesia by using celecoxib combined with tramadol/APAP alleviates post‐operative pain of patients undergoing total knee arthroplasty. Phys Sportsmed, 2017, 45: 316–322. 28475475 10.1080/00913847.2017.1325312

[12] Bedard NA , Pugely AJ , Westermann RW , *et al* Opioid use after total knee arthroplasty: trends and risk factors for prolonged use. J Arthroplasty, 2017, 32: 2390–2394. 28413136 10.1016/j.arth.2017.03.014

[13] Dwyer MK , Tumpowsky CM , Hiltz NL , Lee J , Healy WL , Bedair HS . Characterization of post‐operative opioid use following total joint arthroplasty. J Arthroplasty, 2018, 33: 668–672. 29128235 10.1016/j.arth.2017.10.011

[14] Hadlandsmyth K , Vander Weg MW , McCoy KD , Mosher HJ , Vaughan‐Sarrazin MS , Lund BC . Risk for prolonged opioid use following total knee arthroplasty in veterans. J Arthroplasty, 2018, 33: 119–123. 28927564 10.1016/j.arth.2017.08.022

[15] Smith SR , Bido J , Collins JE , Yang H , Katz JN , Losina E . Impact of preoperative opioid use on total knee arthroplasty outcomes. J Bone Joint Surg Am, 2017, 99: 803–808. 28509820 10.2106/JBJS.16.01200 PMC5426402

[16] Manalo JPM , Castillo T , Hennessy D , Peng Y , Schurko B , Kwon YM . Preoperative opioid medication use negatively affect health related quality of life after total knee arthroplasty. Knee, 2018, 25: 946–951. 30108011 10.1016/j.knee.2018.07.001

[17] Hina N , Fletcher D , Poindessous‐Jazat F , Martinez V . Hyperalgesia induced by low‐dose opioid treatment before orthopaedic surgery: an observational case‐control study. Eur J Anaesthesiol, 2015, 32: 255–261. 25485877 10.1097/EJA.0000000000000197

[18] Trang T , Al‐Hasani R , Salvemini D , Salter MW , Gutstein H , Cahill CM . Pain and poppies: the good, the bad, and the ugly of opioid analgesics. J Neurosci, 2015, 35: 13879–13888. 26468188 10.1523/JNEUROSCI.2711-15.2015 PMC4604226

[19] Weick J , Bawa H , Dirschl DR , Luu HH . Preoperative opioid use is associated with higher readmission and revision rates in total knee and total hip arthroplasty. J Bone Joint Surg Am, 2018, 100: 1171–1176. 30020122 10.2106/JBJS.17.01414

[20] Nguyen LC , Sing DC , Bozic KJ . Preoperative reduction of opioid use before total joint arthroplasty. J Arthroplasty, 2016, 31: 282–287. 27105557 10.1016/j.arth.2016.01.068

[21] Du X , Gu J . The efficacy and safety of parecoxib for reducing pain and opioid consumption following total knee arthroplasty: a meta‐analysis of randomized controlled trials. Int J Surg, 2018, 59: 67–74. 30292001 10.1016/j.ijsu.2018.09.017

[22] Lin J , Zhang L , Yang H . Perioperative administration of selective cyclooxygenase‐2 inhibitors for postoperative pain management in patients after total knee arthroplasty. J Arthroplasty, 2013, 28: 207–213. 22682579 10.1016/j.arth.2012.04.008

[23] Munteanu AM , Cionac Florescu S , Anastase DM , Stoica CI . Is there any analgesic benefit from preoperative vs. postoperative administration of etoricoxib in total knee arthroplasty under spinal anaesthesia?: a randomised double‐blind placebo‐controlled trial. Eur J Anaesthesiol, 2016, 33: 840–845. 27454662 10.1097/EJA.0000000000000521

[24] Zhu YZ , Yao R , Zhang Z , Xu H , Wang LW . Parecoxib prevents early postoperative cognitive dysfunction in elderly patients undergoing total knee arthroplasty: a double‐blind, randomized clinical consort study. Medicine (Baltimore), 2016, 95: e4082. 27428192 10.1097/MD.0000000000004082 PMC4956786

[25] Zhu Y , Yao R , Li Y , *et al* Protective effect of celecoxib on early postoperative cognitive dysfunction in geriatric patients. Front Neurol, 2018, 9: 633. 30131758 10.3389/fneur.2018.00633 PMC6090028

[26] Greimel F , Maderbacher G , Baier C , *et al* Matched‐pair analysis of local infiltration analgesia in total knee arthroplasty: patient satisfaction and perioperative pain management in 846 cases. J Knee Surg, 2018 10.1055/s-0038-1672156. [Epub ahead of print] 30292173

[27] Zhang Z , Shen B . Effectiveness and weakness of local infiltration analgesia in total knee arthroplasty: a systematic review. J Int Med Res, 2018, 46: 4874–4884. 30318966 10.1177/0300060518799616 PMC6300945

[28] Zhang LK , Ma JX , Kuang MJ , Ma XL . Comparison of periarticular local infiltration analgesia with femoral nerve block for total knee arthroplasty: a meta‐analysis of randomized controlled trials. J Arthroplasty, 2018, 33: 1972–1978. 29455938 10.1016/j.arth.2017.12.042

[29] Wall PDH , Parsons NR , Parsons H , *et al* A pragmatic randomised controlled trial comparing the efficacy of a femoral nerve block and periarticular infiltration for early pain relief following total knee arthroplasty. Bone Joint J, 2017, 99‐B: 904–911. 10.1302/0301-620X.99B7.BJJ-2016-0767.R2 PMC5633832 28663395

[30] Hu B , Lin T , Yan SG , *et al* Local infiltration analgesia versus regional blockade for postoperative analgesia in total knee arthroplasty: a meta‐analysis of randomized controlled trials. Pain Physician, 2016, 19: 205–214. 27228509

[31] Sardana V , Burzynski JM , Scuderi GR . Adductor canal block or local infiltrate analgesia for pain control after total knee arthroplasty? A systematic review and meta‐analysis of randomized controlled trials. J Arthroplasty, 2019, 34: 183–189. 30360981 10.1016/j.arth.2018.09.083

[32] Snyder MA , Scheuerman CM , Gregg JL , Ruhnke CJ , Eten K . Improving total knee arthroplasty perioperative pain management using a periarticular injection with bupivacaine liposomal suspension. Arthroplast Today, 2016, 2: 37–42. 28326395 10.1016/j.artd.2015.05.005 PMC4957154

[33] Heim EA , Grier AJ , Butler RJ , Bushmiaer M , Queen RM , Barnes CL . Use of liposomal bupivacaine instead of an epidural can improve outcomes following total knee arthroplasty. J Surg Orthop Adv, 2015, 24: 230–234. 26731386

[34] Alijanipour P , Tan TL , Matthews CN , *et al* Periarticular injection of liposomal bupivacaine offers no benefit over standard bupivacaine in total knee arthroplasty: a prospective, randomized, controlled trial. J Arthroplasty, 2017, 32: 628–634. 27667533 10.1016/j.arth.2016.07.023

[35] Mont MA , Beaver WB , Dysart SH , Barrington JW , Del Gaizo DJ . Local infiltration analgesia with liposomal bupivacaine improves pain scores and reduces opioid use after total knee arthroplasty: results of a randomized controlled trial. J Arthroplasty, 2018, 33: 90–96. 28802777 10.1016/j.arth.2017.07.024

[36] Talmo CT , Kent SE , Fredett AN , Anderson MC , Hassan MK , Mattingly DA . Prospective randomized trial comparing femoral nerve block with intraoperative local anesthetic injection of liposomal bupivacaine in total knee arthroplasty. J Arthroplasty, 2018, 33: 3474–3478. 30150152 10.1016/j.arth.2018.07.018

[37] Seangleulur A , Vanasbodeekul P , Prapaitrakool S , *et al* The efficacy of local infiltration analgesia in the early postoperative period after total knee arthroplasty: a systematic review and meta‐analysis. Eur J Anaesthesiol, 2016, 33: 816–831. 27428259 10.1097/EJA.0000000000000516

[38] Monahan A , Guay J , Hajduk J , Suresh S . Regional analgesia added to general anesthesia compared with general anesthesia plus systemic analgesia for cardiac surgery in children: a systematic review and meta‐analysis of randomized clinical trials. Anesth Analg, 2019, 128: 130–136. 30300178 10.1213/ANE.0000000000003831

[39] Treschan TA , Taguchi A , Ali SZ , *et al* The effects of epidural and general anesthesia on tissue oxygenation. Anesth Analg, 2003, 96: 1553–1557. 12760974 10.1213/01.ANE.0000063824.43113.DB

[40] Pugely AJ , Martin CT , Gao Y , Mendoza‐Lattes S , Callaghan JJ . Differences in short‐term complications between spinal and general anesthesia for primary total knee arthroplasty. J Bone Joint Surg Am, 2013, 95: 193–199. 23269359 10.2106/JBJS.K.01682

[41] Park YB , Chae WS , Park SH , Yu JS , Lee SG , Yim SJ . Comparison of short‐term complications of general and spinal anesthesia for primary unilateral total knee arthroplasty. Knee Surg Relat Res, 2017, 29: 96–103. 28545173 10.5792/ksrr.16.009 PMC5450584

[42] Mahan MC , Jildeh TR , Tenbrunsel TN , Davis JJ . Mepivacaine spinal anesthesia facilitates rapid recovery in total knee arthroplasty compared to bupivacaine. J Arthroplasty, 2018, 33: 1699–1704. 29429882 10.1016/j.arth.2018.01.009

[43] Block BM , Liu SS , Rowlingson AJ , Cowan AR , Cowan JA Jr , Wu CL . Efficacy of postoperative epidural analgesia: a meta‐analysis. Jama, 2003, 290: 2455–2463. 14612482 10.1001/jama.290.18.2455

[44] Koh JC , Song Y , Kim SY , Park S , Ko SH , Han DW . Postoperative pain and patient‐controlled epidural analgesia‐related adverse effects in young and elderly patients: a retrospective analysis of 2,435 patients. J Pain Res, 2017, 10: 897–904. 28442931 10.2147/JPR.S133235 PMC5396922

[45] Fedriani de Matos JJ , Atienza Carrasco FJ , Diaz Crespo J , Moreno Martin A , Tatsidis Tatsidis P , Torres Morera LM . Effectiveness and safety of continuous ultrasound‐guided femoral nerve block versus epidural analgesia after total knee arthroplasty. Rev Esp Anestesiol Reanim, 2017, 64: 79–85. 27400891 10.1016/j.redar.2016.05.008

[46] Kasture S , Saraf H . Epidural versus intra‐articular infusion analgesia following total knee replacement. J Orthop Surg (Hong Kong), 2015, 23: 287–289. 26715701 10.1177/230949901502300304

[47] Li C , Qu J , Pan S , Qu Y . Local infiltration anesthesia versus epidural analgesia for postoperative pain control in total knee arthroplasty: a systematic review and meta‐analysis. J Orthop Surg Res, 2018, 13: 112. 29769140 10.1186/s13018-018-0770-9 PMC5956819

[48] Fowler SJ , Symons J , Sabato S , Myles PS . Epidural analgesia compared with peripheral nerve blockade after major knee surgery: a systematic review and meta‐analysis of randomized trials. Br J Anaesth, 2008, 100: 154–164. 18211990 10.1093/bja/aem373

[49] Dixit V , Fathima S , Walsh SM , *et al* Effectiveness of continuous versus single injection femoral nerve block for total knee arthroplasty: a double blinded, randomized trial. Knee, 2018, 25: 623–630. 29705075 10.1016/j.knee.2018.04.001

[50] Chan EY , Fransen M , Parker DA , Assam PN , Chua N . Femoral nerve blocks for acute postoperative pain after knee replacement surgery. Cochrane Database Syst Rev, 2014, 13: CD009941. 10.1002/14651858.CD009941.pub2 PMC7173746 24825360

[51] Charous MT , Madison SJ , Suresh PJ , *et al* Continuous femoral nerve blocks: varying local anesthetic delivery method (bolus versus basal) to minimize quadriceps motor block while maintaining sensory block. Anesthesiology, 2011, 115: 774–781. 21394001 10.1097/ALN.0b013e3182124dc6 PMC3116995

[52] Li D , Ma GG . Analgesic efficacy and quadriceps strength of adductor canal block versus femoral nerve block following total knee arthroplasty. Knee Surg Sports Traumatol Arthrosc, 2016, 24: 2614–2619. 26611901 10.1007/s00167-015-3874-3

[53] Karkhur Y , Mahajan R , Kakralia A , Pandey AP , Kapoor MC . A comparative analysis of femoral nerve block with adductor canal block following total knee arthroplasty: a systematic literature review. J Anaesthesiol Clin Pharmacol, 2018, 34: 433–438. 30774223 10.4103/joacp.JOACP_198_18 PMC6360900

[54] Tan Z , Kang P , Pei F , Shen B , Zhou Z , Yang J . A comparison of adductor canal block and femoral nerve block after total‐knee arthroplasty regarding analgesic effect, effectiveness of early rehabilitation, and lateral knee pain relief in the early stage. Medicine (Baltimore), 2018, 97: e13391. 30508936 10.1097/MD.0000000000013391 PMC6283224

[55] Shah NA , Jain NP , Panchal KA . Adductor canal blockade following total knee arthroplasty‐continuous or single shot technique? Role in postoperative analgesia, ambulation ability and early functional recovery: a randomized controlled trial. J Arthroplasty, 2015, 30: 1476–1481. 25824025 10.1016/j.arth.2015.03.006

[56] Rantasalo MT , Palanne R , Juutilainen K , *et al* Randomised controlled study comparing general and spinal anaesthesia with and without a tourniquet on the outcomes of total knee arthroplasty: study protocol. BMJ Open, 2018, 8: e025546. 10.1136/bmjopen-2018-025546 PMC6307602 30580277

[57] Yik JH , Tham WYW , Tay KH , Shen L , Krishna L . Perioperative pregabalin does not reduce opioid requirements in total knee arthroplasty. Knee Surg Sports Traumatol Arthrosc, 2019, 27: 2104–2110. 30739128 10.1007/s00167-019-05385-7

[58] Ryu JH , Jeon YT , Min B , Hwang JY , Sohn HM . Effects of palonosetron for prophylaxis of postoperative nausea and vomiting in high‐risk patients undergoing total knee arthroplasty: a prospective, randomized, double‐blind, placebo‐controlled study. PLoS One, 2018, 13: e0196388. 29758039 10.1371/journal.pone.0196388 PMC5951557

[59] Borckardt JJ , Reeves ST , Milliken C , *et al* Prefrontal versus motor cortex transcranial direct current stimulation (tDCS) effects on post‐surgical opioid use. Brain Stimul, 2017, 10: 1096–1101. 28917592 10.1016/j.brs.2017.09.006 PMC5675751

[60] Song MH , Kim BH , Ahn SJ , *et al* Peri‐articular injections of local anaesthesia can replace patient‐controlled analgesia after total knee arthroplasty: a randomised controlled study. Int Orthop, 2016, 40: 295–299. 26227922 10.1007/s00264-015-2940-2

[61] Walder B , Schafer M , Henzi I , Tramèr MR . Efficacy and safety of patient‐controlled opioid analgesia for acute postoperative pain. A quantitative systematic review. Acta Anaesthesiol Scand, 2001, 45: 795–804. 11472277 10.1034/j.1399-6576.2001.045007795.x

[62] Dias AS , Rinaldi T , Barbosa LG . The impact of patients controlled analgesia undergoing orthopedic surgery. Braz J Anesthesiol, 2016, 66: 265–271. 27108823 10.1016/j.bjane.2013.06.023

[63] Novello‐Siegenthaler A , Hamdani M , Iselin‐Chaves I , Fournier R . Ultrasound‐guided continuous femoral nerve block: a randomized trial on the influence of femoral nerve catheter orifice configuration (six‐hole versus end‐hole) on post‐operative analgesia after total knee arthroplasty. BMC Anesthesiol, 2018, 18: 191. 30567487 10.1186/s12871-018-0648-8 PMC6300902

[64] Kim DH , Beathe JC , Lin Y , *et al* Addition of infiltration between the popliteal artery and the capsule of the posterior knee and adductor canal block to periarticular injection enhances postoperative pain control in total knee arthroplasty: a randomized controlled trial. Anesth Analg, 2019, 129: 526–535. 30234517 10.1213/ANE.0000000000003794

[65] Tong QJ , Lim YC , Tham HM . Comparing adductor canal block with local infiltration analgesia in total knee arthroplasty: a prospective, blinded and randomized clinical trial. J Clin Anesth, 2018, 46: 39–43. 29414612 10.1016/j.jclinane.2018.01.014

[66] McCarthy D , McNamara J , Galbraith J , Loughnane F , Shorten G , Iohom G . A comparison of the analgesic efficacy of local infiltration analgesia vs. intrathecal morphine after total knee replacement: a randomised controlled trial. Eur J Anaesthesiol, 2019, 36: 264–271. 30640244 10.1097/EJA.0000000000000943

[67] Koniuch KL , Buys MJ , Campbell B , *et al* Serum ropivacaine levels after local infiltration analgesia during total knee arthroplasty with and without adductor canal block. Reg Anesth Pain Med, 2019: pii: rapm‐2018‐100043 10.1136/rapm-2018-100043. [Epub ahead of print] 30635510

